# Long-Term Follow-Up of Lentigo Maligna Patients Treated with Imiquimod 5% Cream

**DOI:** 10.3390/cancers15051546

**Published:** 2023-02-28

**Authors:** S. Morteza Seyed Jafari, Flavia Folini-Huesser, Simone Cazzaniga, Robert E. Hunger

**Affiliations:** Department of Dermatology, Inselspital, Bern University Hospital, University of Bern, 3012 Bern, Switzerland

**Keywords:** imiquimod therapy, lentigo maligna, non-invasive therapy

## Abstract

**Simple Summary:**

The study aimed to investigate the long-term efficacy of imiquimod 5% cream for lentigo maligna, with a focus on disease recurrence and the possible prognostic factors of disease-free survival. If surgical excision is not possible due to the patients’ age/comorbidities or critical cosmetic localization, imiquimod provides promising outcomes with an optimal risk of relapse for the management of LM.

**Abstract:**

Background: The study investigated the long-term efficacy of imiquimod 5% cream for LM, with a focus on disease recurrence and the possible prognostic factors of disease-free survival (DFS) in a cohort, with long-term follow-up. Methods: Consecutive patients with histologically confirmed LM were included. Imiquimod 5% cream was applied until weeping erosion appeared on the LM-affected skin. The evaluation was performed through clinical examination and dermoscopy. Results: We analyzed 111 patients with LM (median age: 72 years, 61.3% women) with tumor clearance after imiquimod therapy, with a median follow-up of 8 years. The overall patient survival rates were 85.5% (95% confidence interval (CI): 78.5–92.6) and 70.4% (95% CI: 60.3–80.5) at 5 and 10 years, respectively. Among the 23 patients (20.1%) with relapse at follow-up, 17 (73.9%) were treated with surgery, five (21.7%) continued imiquimod therapy, and one (4.3%) underwent both surgery and radiotherapy. After adjustment for age and LM area in multivariable models, localization of LM in the nasal region was identified as a prognostic factor for DFS (HR = 2.66; 95% CI: 1.06–6.64). Conclusion: If surgical excision is not possible due to the patients’ age/comorbidities or critical cosmetic localization, imiquimod could provide optimal outcomes with an optimal risk of relapse for the management of LM.

## 1. Introduction

Lentigo maligna (LM), the most common melanoma in situ, occurs in elderly patients with chronic sun-exposed skin [1]. Approximately 5–15% of all LM can transform into an invasive lentigo maligna melanoma over years, therefore it is important to treat LM and prevent progression of disease [2,3]. Once LM is diagnosed, surgical excision is the standard treatment of choice [4]. Nevertheless, surgical treatment cannot be performed in all cases [3]. Therefore, in some patients, due to comorbidities or in esthetically challenging localizations, alternative non-surgical treatment options, such as radiotherapy [5], cryotherapy [6] and laser [7], may be considered for LM [3]. In recent studies, imiquimod 5% cream (Aldara^®^), has been reported as an appropriate non-surgical alternative to treat LM [3,8]. Furthermore, imiquimod has been recommended as a (neo-)adjuvant therapy to surgical intervention [9]. Imiquimod, an imidazoquinoline amine, is an immune-response modifier that was primarily approved for treating external genital and perianal warts [8]. Imiquimod activates the innate immune system, resulting in a cytokine release and costimulatory molecule expression, followed by T-cell activation. Studies using imiquimod as a treatment for a variety of benign, premalignant and malignant diseases, show promise [8].

The current study aimed to investigate the response of LM to imiquimod 5% cream as a primary therapy, in a cohort of patients with long-term follow-up, with a focus on disease recurrence and possible prognostic factors of disease-free survival (DFS).

## 2. Materials and Methods

### 2.1. Study Population

From 2003 to 2020, all consecutive patients with a histologically confirmed diagnosis of LM, who had undergone therapy with imiquimod 5% cream (MEDA Pharma GmbH, Bad Homburg, Germany), were retrospectively included in the study. Patients, who did not respond to therapy, i.e., did not show proper/wanted local reaction, were excluded. The patients signed an informed consent form, and the study was conducted in accordance with the standards of human experimentation and the Declaration of Helsinki of 1975, as revised in 1983 and approved by the Ethical Committee of the Canton of Bern (KEK-BE: 382/2016).

### 2.2. Collected Data

The following patient characteristics were collected at the start of treatment: sex, age, Fitzpatrick skin type, tumor localization and size, number of daily applications, duration of the treatment, therapy reactions, side effects, occurrence of secondary melanoma, LM recurrence, and patient death.

### 2.3. Procedures

After obtaining the baseline clinical and dermoscopic images, a biopsy was performed from the lesion to confirm the clinical diagnosis. Imiquimod local therapy was initiated when the diagnosis of LM was confirmed. The therapy consisted of one to two daily applications of imiquimod cream. Therapy was administered to all patients until weeping erosion was observed in the treated skin area. All patients underwent regular clinical follow-ups during and after imiquimod treatment, as already discussed elsewhere [3,10]. In case of clinical suspicion of recurrence during the follow-ups, in vivo reflectance confocal microscopy (RCM) was performed [3]. Upon the presence of clues for recurrences in RCM, a histological examination was performed. In addition, for some patients, RCM was used as quality control.

### 2.4. Outcomes

The main study outcome was LM-related patient survival. The secondary outcomes included DFS in terms of LM relapse and the overall patient survival rate. The factors associated with DFS were also explored.

### 2.5. Statistical Analysis

For descriptive purposes, continuous data were presented as medians with ranges, while categorical data have been presented as numbers with percentages. For the purpose of analysis, continuous data were categorized using clinically relevant cutoff points. The Kaplan–Meier method was used to calculate survival estimates, along with their 95% confidence intervals (CI). An exact 95% CI was employed in the case of zero-events [11]. Uni- and multivariable Cox regression analyses, including the age and lesion area as adjustment factors, were used to assess the factors associated with DFS. All tests were considered statistically significant at *p* < 0.05. Analyses were performed using SPSS v.26.0 (IBM Corp, Armonk, NY, USA) and the R software v.4.1.1 (R Foundation for Statistical Computing, Vienna, Austria).

## 3. Results

**Figure 1:**
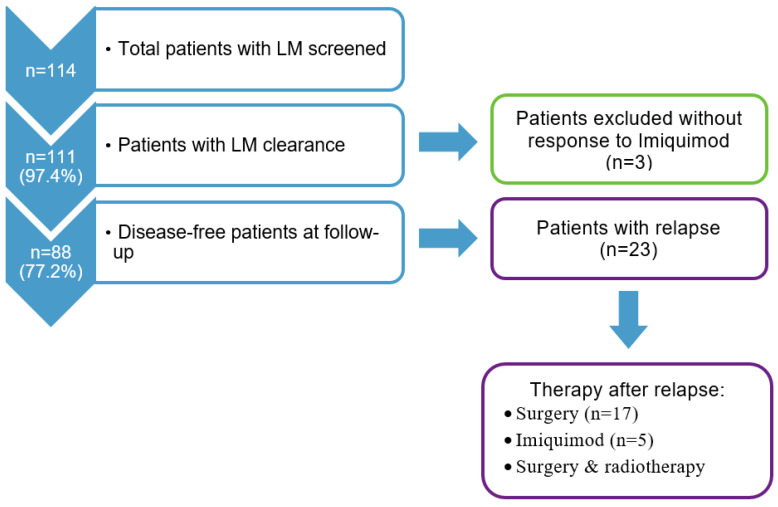
Flowchart of the study cohort selection and follow-up.

A flowchart of the study cohort selection is shown in Figure 1. Of the 114 patients with a first diagnosis of LM, 111 patients (median age: 72 years, 61.3% women) with tumor clearance after imiquimod local therapy were included in our analysis.

### 3.1. Characteristics of the Study Cohort

The demographic and clinical characteristics of the study population are shown in Table 1. The majority of the patients had Fitzpatrick skin types II (60.0%) or III (30.9%). LM was mainly located in the cheeks (44.1%), nasal region (28.8%), and the temporal/auricular region (12.6%). The median major diameter of the lesions was 18 mm, ranging from 5 to 60 mm, and the median equivalent area was 1.7 cm^2^, ranging from 0.1 to 19.6 cm^2^.

### 3.2. Imiquimod Therapy

Imiquimod 5% cream was applied for a median of 4.1 weeks (range: 1.1–55.3 weeks), with two applications/day (range: 0.7–2.0). Most patients (85.6%) had erosions, oozing, or eschars as a reaction to therapy, while erythema or inflammation was reported in 12.6% of patients (Table 2).

### 3.3. Follow-up and Survival

**Figure 2:**
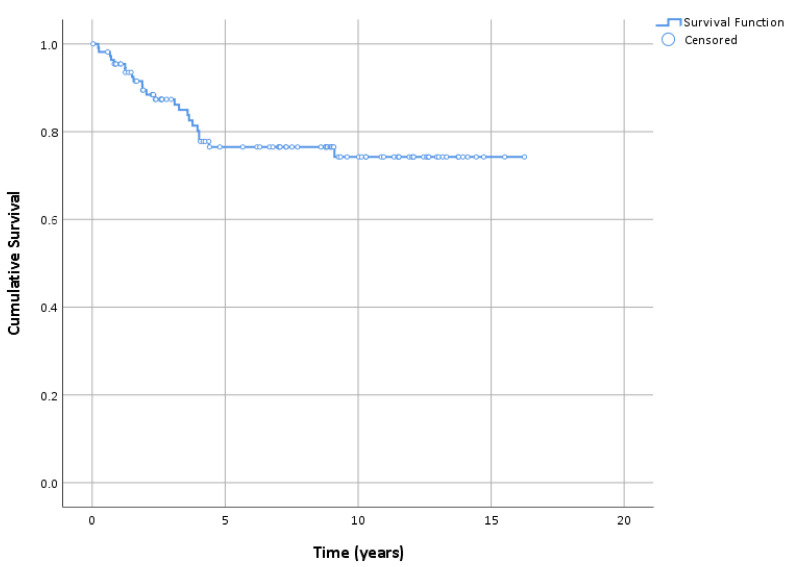
Kaplan–Meier plot of disease-free survival.

**Figure 3:**
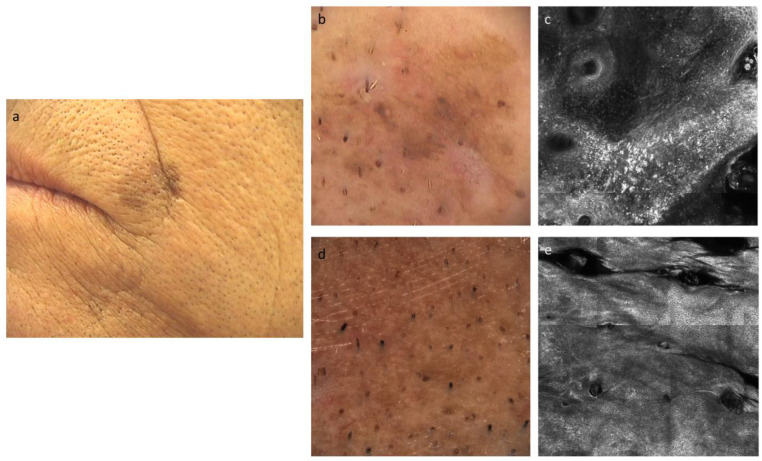
(**a**) Clinical images of lentigo maligna near the lip of a 69 y/o male, at the time of diagnosis. (**b**) Dermoscopic image of lentigo maligna before the therapy, which shows hyperpigmented follicular opening, with some obliterated hair follicles. (**c**) Confocal microscopy image of lentigo maligna before the therapy, which shows the loss of the epidermic architecture (disrupted honeycomb) with the presence of atypical melanocytes. (**d**,**e**) Dermoscopic and confocal microscopic images of the treated area, which show regular and symmetric patterns, without any signs of atypia.

The patients were followed-up for a median of 8 years (range: 15–16.8 years). The overall patient survival rates were 85.5% (95% CI: 78.5–92.6) and 70.4% (95% CI: 60.3–80.5) at 5 and 10 years, respectively (Table 3). However, when considering the specific causes, we did not observe any LM-related deaths during the follow-up period. Based on the exact estimates, with the 95% confidence, we can say that the LM-related survival was >95% at 5 years and >90% at 10 years. Regarding relapses, the DFS was 87.4% (95% CI: 80.9–93.8), 76.5% (95% CI: 67.8–85.2), and 74.3% (95% CI: 64.8–83.8) at 3, 5, and 10 years, respectively (Figure 2 and Figure 3). Among the 23 patients (20.1%) who experienced relapse, 17 (73.9%) were treated surgically, five (21.7%) continued imiquimod therapy and one (4.3%) underwent both surgery and radiotherapy (Figure 1). Among the patients who experienced relapse and were treated surgically, in five patients, an invasive component was found in the excised tissue. Except in one case (Breslow index 2 mm), none of the cases showed an invasive component thicker than 0.8 mm (Breslow index 0.1–0.5 mm). Regression and ulceration were not reported in these cases. The patients were managed according to the guidelines. A second malignant melanoma was reported in 16 (14.4%) patients.

### 3.4. Prognostic Factors for DFS

Table 4 presents the analyses of selected factors associated with DFS. No factor was significantly associated in the univariate analysis. However, when adjusting for the patients’ age and LM area in multivariable models, localization of LM in the nasal region was identified as a risk factor for DFS (HR = 2.66; 95% CI: 1.06–6.64).

## 4. Discussion

LM is a melanocytic neoplasm that is more common in elderly patients relative to other melanoma subtypes (nodular, superficial spreading, and acral) and characterized by a prolonged radial growth phase. By definition, LM is defined as melanoma in situ, and is thus confined to the epidermis [9,12]. Evidence suggests that rates of LM and LMM are increasing faster than any other melanoma subtypes [12]. Nevertheless, it is not clear, if it is the result of increased awareness and surveillance or if the data indicate a true increase in incidence [12]. In spite of the relatively low progression rate of LM into invasive melanoma, it is important to treat LM and prevent the progression of disease [3,13]. Surgical excision is the gold standard treatment of LM, since it allows for the confirmation of histologically negative margins and results in lower recurrence rates [3,12]. However, surgical removal often causes substantial morbidity, since lesions of LM are typically large and most commonly occur in cosmetically sensitive areas [9]. Consequently, less invasive treatment alternatives have been therefore considered [7]. Use of radiation therapy, as one of such therapies, has been extensively reported in the management of patients with of LM, especially for the treatment of LM, where surgical resection would produce poor functional or cosmetic outcomes [14,15,16]. Radiotherapy has good tolerance and generally favorable cosmetic results, but might increase risk of squamous cell carcinoma [12]. Cryotherapy has also been used to treat LM, as melanocytes are susceptible to cold-induced damage. This method is easy to apply and fast, but healing time could be longer and scarring may occur [12]. Furthermore, it has been reported that ablative laser treatment (such as Er:YAG lasers and carbon dioxide) of LM involves the induction of energized light waves of varying frequencies targeted to lesions of concern. [14,15,16]. Early reports of laser treatments of LM suggested superior rapid treatment time, cosmetic outcomes, improved tolerability and reduced post-treatment care requirements [14]. Nevertheless, subsequent research has consistently shown poor complete response rates and high recurrence rates, secondary to difficulties with adequate margin clearance and lesion targeting [14]. Electrodesiccation and curettage, photodynamic therapy, and chemical peels have also been proposed and studied for the treatment of LM [12,15,16].

In recent years, topical imiquimod, as a topical immunomodulating agent, has proven a good therapeutic alternative to surgical excision in patients with comorbidities, or who are at risk of cosmetic disfigurement [3,17]. The therapy works through the Toll-like receptor signaling pathway, inducing the secretion of various pro-inflammatory cytokines, and activates immune cells, such as monocytes and T cells [18]. In the treatment of LM, it is presumed that imiquimod induces a cytotoxic T-cell-mediated immune response, which elicits the destruction of malignant melanocytes [3,19]. Topical imiquimod 5% cream has been investigated as off-label primary or adjuvant treatment for melanoma in situ, lentigo maligna type (LM) [20]. The most important question concerns the long-term outcomes for patients treated with imiquimod cream [9]. To our knowledge, the current study has one of the largest cohorts of LM patients primarily treated with imiquimod 5% cream, with an optimal long-term follow-up. The study revealed an excellent therapeutic response, with a promising recurrence rate after a median follow-up of 8 years. Based on the exact estimates, we can affirm that LM-related survival was >95% at 5 years and >90% at 10 years, with a CI of 95%. Moreover, the DFS was 87.4%, 76.5%, and 74.3% at 3, 5, and 10 years, respectively. Recent studies investigating the efficacy of imiquimod in the treatment of LM have suggested long-term clearance rates as high as 50% to 88% [14,21]. The mean recurrence rate calculated from the pooled data in a recent systematic review was 24.5%, with a heterogeneity of I2 = 62.6% [14]. When adjusting for the patients’ age and LM area in multivariable models, localization of LM in the nasal region was identified as a prognostic risk factor for DFS. This may be possible because of the difficulty in the proper application of the cream in this area, since the tumor needs to be properly treated to obtain a complete local response, as reported by Naylor et al. [22] Powell et al. could not identify the histological features of prognostic significance [21]. However, the ability to develop an inflammatory reaction to imiquimod was a strong predictor of therapeutic benefits in similar studies [21]. It has been shown how essential it is that the patient develops an inflammatory reaction for the treatment to be successful [23]. In a similar study, LM persisted in 100% of the patients who failed to develop an inflammatory reaction, and in 57% of those with a mild reaction, but only in 27% of those with a brisk reaction [23]. The mechanism of this lack of response is unknown. Increased concentrations of regulatory T cells in lesions may be important [23,24]. Alternatively, patients with LM who are non-responders may have genetic polymorphisms that influence the secretion of interleukin-10, an inhibitory cytokine that slows the generation of CD4 and CD8 effector T cells, which are able to kill tumor cells [23,25].

Among the patients who experienced relapse and were treated surgically, in five patients, an invasive component was found in the excised tissue. The most obvious explanation is the progression of the lentigo maligna to an invasive melanoma. In a recent review, a progression of LM to LMM was observed in 1.8% of the patients, with an average of 3.9 months (range 0–11 months) after completion of treatment [26]. Another explanation, however, is an inaccurate diagnosis due to a sampling error, since only a punch-biopsy might not be representative for the diagnosis of whole lesion. As shown before [27], histopathologic misdiagnosis is more common for melanomas that have been assessed with punch and shave biopsy than with excisional biopsy. This issue has recently raised concerns regarding the therapy of imiquimod 5% cream for LM. The first issue is that the entire lesion is not available for histological analysis and the biopsies could be subject to a sampling error [21]. Another concern was whether clinically resolved lesions following imiquimod treatment may still harbor the foci of LM or LMM histologically [9,14].

## 5. Conclusions

Imiquimod is an immune-response modifier that stimulates both the innate and adaptive arms of the immune system, resulting in an enhanced immune function [3,8]. Despite the existing mentioned concerns and limitations of the study (i.e., retrospective design, and therefore lack of a control group), imiquimod cream has been consistently reported as a safe and well-tolerated treatment alternative, capable of achieving excellent cosmetic and clinical responses [14], especially in patients ineligible for surgical intervention due to comorbidities, tumor size, or cosmetic reasons. Compared to surgery, it offers a cheap and painless therapy, with a low morbidity and reasonable efficacy [3]. Therefore, as shown in our study with long-term patient follow-up, this local therapy may be a reasonable alternative modality for the management of LM, as opposed to more aggressive therapies. However, a head-to-head clinical trial is required to provide accurate data on the long-term efficacy and recurrence rate of imiquimod cream compared to surgical excision as a standard method of choice.

## Figures and Tables

**Table 1:** Demographics and clinical characteristics of the study population.

		*n* = 111 *
Age (years)	Median, range	72.0 (38.0–93.0)
	<70.0	42 (37.8%)
	70.0–79.9	41 (36.9%)
	80.0+	28 (25.2%)
Sex	Male	43 (38.7%)
	Female	68 (61.3%)
Fitzpatrick skin type	I	10 (9.1%)
	II	66 (60.0%)
	III	34 (30.9%)
Localization **	Cheek	49 (44.1%)
	Nasal region	32 (28.8%)
	Temporal/auricular region	14 (12.6%)
	Frontal region	6 (5.4%)
	Orbital region	5 (4.5%)
	Other face/head areas	3 (2.7%)
	Other locations	5 (4.5%)
Major diameter (mm)	Median, range	18.0 (5.0–60.0)
	<10.0	17 (15.5%)
	10.0–19.0	40 (36.4%)
	20.0+	53 (48.2%)
Area (cm^2^) ***	Median, range	1.7 (0.1–19.6)
	<1.0	42 (38.2%)
	1.0–2.9	30 (27.3%)
	3.0+	38 (34.5%)

* Numbers may not add up to the total due to missing data; ** Multiple locations per patient are possible; *** Area of the equivalent ellipse.

**Table 2:** Imiquimod therapy in the study population (*n* = 111).

Duration of application (weeks)	Median, range	4.1 (1.1–55.3)
	<3.0	36 (32.4%)
	3.0–5.9	33 (29.7%)
	6.0+	42 (37.8%)
No. applications/day	Median, range	2.0 (0.7–2.0)
	≤1	43 (38.7%)
	>1	68 (61.3%)
Maximal reaction	None	2 (1.8%)
	Skin redness	14 (12.6%)
	Erosions, oozing, or eschars	95 (85.6%)

**Table 3:** Kaplan–Meier estimates of disease-free, overall, and lentigo maligna-related survival in the study population.

	N Events, Survival (95% CI)		
	3 Years	5 Years	10 Years
LM relapse	13, 87.4% (80.9–93.8)	22, 76.5% (67.8–85.2)	23, 74.3% (64.8–83.8)
Overall death	8, 92.1% (86.9–97.4)	14, 85.5% (78.5–92.6)	25, 70.4% (60.3–80.5)
LM-related death	0, 100% (95.9–100)	0, 100% (95.1–100)	0, 100% (90.5–100)

CI: Confidence interval, LM: Lentigo maligna.

**Table 4:** Analysis of selected factors associated with disease-free survival.

			Univariate Analysis			Multivariable Analysis	
			N Events	10 y Survival *	*p* **	HR (95% CI)	*p* ***
Age (years)	<70.0		4	82.3%	0.20	1	
	70.0–79.9		16	68.1%		2.17 (0.88–5.37)	0.09
	80.0+		3	83.4%		0.99 (0.26–2.79)	0.99
Sex	Male		8	73.0%	0.62	1	
	Female		15	74.6%		1.51 (0.62–3.63)	0.36
Fitzpatrick skin type	I		1	88.9%	0.42	1	
	II		12	77.9%		1.69 (0.21–13.45)	0.62
	III		10	64.4%		2.47 (0.31–20.06)	0.40
Localization	Cheek	No	14	71.9%	0.60	1	
		Yes	9	77.5%		0.92 (0.38–2.21)	0.85
	Nasal region	No	14	79.7%	0.09	1	
		Yes	9	57.6%		2.66 (1.06–6.64)	0.04
	Temporal/auricular region	No	20	74.2%	0.99	1	
		Yes	3	74.6%		0.80 (0.23–2.77)	0.73
	Other face/head areas	No	20	74.0%	0.98	1	
		Yes	3	75.7%		0.84 (0.25–2.83)	0.77
Major diameter (mm)	<10.0		4	75.5%	0.71	1	
	10.0–19.0		10	71.2%		1.12 (0.35–3.60)	0.84
	20.0+		9	75.6%		0.75 (0.22–2.49)	0.64
Area (cm^2^)	<1.0		8	78.8%	0.10	1	
	1.0–2.9		11	56.4%		1.89 (0.76–4.70)	0.17
	3.0+		4	86.1%		0.53 (0.16–1.77)	0.30
Duration of imiquimod application (weeks)	<3.0		8	70.3%	0.88	1	
	3.0–5.9		6	79.6%		0.77 (0.27–2.24)	0.63
	6.0+		9	74.4%		0.81 (0.30–2.16)	0.68
No. applications/day	≤1		9	75.0%	0.87	1	
	>1		14	74.2%		1.03 (0.44–2.43)	0.94
Maximal reaction	None/skin redness		3	76.2%	0.62	1	
	Erosions, oozing or eschars		20	74.8%		1.54 (0.45–5.24)	0.49

CI: Confidence interval, HR: Hazard ratio; * Kaplan–Meier cumulative survival estimate; ** Log-rank test; *** Multivariable Cox regression models, including age and lesion area as adjustment factors. When assessing the major diameter, the lesion area was not included as a covariate.

## Data Availability

All relevant data were presented in the current paper.
